# Insecticides outweigh rye cover crop in triggering secondary pest outbreaks

**DOI:** 10.1002/ps.70109

**Published:** 2025-08-02

**Authors:** Zeus Mateos‐Fierro, Ashley Leach, Ian Kaplan

**Affiliations:** ^1^ Department of Entomology Purdue University West Lafayette IN USA; ^2^ Department of Entomology Ohio State University Wooster OH USA

**Keywords:** agricultural inputs, biological control, broad‐spectrum insecticides, integrated pest management, natural enemies, threshold

## Abstract

**BACKGROUND:**

Broad‐spectrum insecticides are extensively used to control pests and secure crop yields. However, insecticides may have unintended effects on natural enemies (e.g., predators and parasitoid wasps) and secondary pests. Cover crops can also be used as part of integrated pest management (IPM) programs to protect natural enemies and avoid secondary pest outbreaks. Yet, some pests can use cover crops as green bridges to colonize the crop. We explored the use of rye in commercial watermelon fields, with three intensity levels employed by growers: (i) no rye, (ii) 1:3 rye‐watermelon ratio and (iii) 1:1 rye‐watermelon ratio. Then, we tested the combined effects of insecticide programs (conventional *vs.* threshold‐based recommendations) and rye use (present *vs.* absent) at three research farms over 2 years.

**RESULTS:**

In both the on‐farm survey and research‐plot trial, rye weakly affected arthropods, with no impacts on cucumber beetles or natural enemies and marginally positive and negative effects on spider mites and melon aphids, respectively. In contrast, conventional insecticide programs in the research‐plot trial effectively controlled the main watermelon pest (striped cucumber beetle) but triggered 45.5 and 21.2% population increases in spider mite and melon aphid, respectively, while natural enemies decreased by 82.3% compared to threshold recommendations. Watermelon yield and quality were generally unaffected by treatments.

**CONCLUSION:**

We show that insecticide practices are more relevant in shaping arthropod dynamics than cover crops in watermelon. Our study highlights the importance of IPM to protect natural enemies, prevent pest outbreaks, minimize costs on unnecessary insecticide applications and enhance sustainable agriculture. © 2025 The Author(s). *Pest Management Science* published by John Wiley & Sons Ltd on behalf of Society of Chemical Industry.

## Graphical abstract

Insecticides outweigh rye cover crop in triggering secondary pest outbreaks. Zeus Mateos‐Fierro*, Ashley Leach, Ian Kaplan. Spider mite and melon aphid outbreaks are enhanced with broad‐spectrum insecticides, while natural enemies are reduced. However, rye cover crop has little effect on either pest or natural enemy populations.

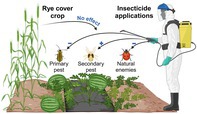

## 1 INTRODUCTION

Chemical insecticides are extensively used by many farmers, but their overuse causes unintended, detrimental effects on beneficial insects such as natural enemies (e.g., predators and parasitoid wasps).^1^, ^2^, ^3^ In particular, broad‐spectrum insecticides, including pyrethroids and neonicotinoids, pose the greatest threat, despite serving as the predominant insecticides used in major cropping systems like cucurbits,^4^ berries^5^ and fruit trees.^6^ Broad‐spectrum insecticides reduce natural enemy populations (i.e., lethal effects)^7^, ^8^ and/or indirectly impair their reproduction and behavior (i.e., sub‐lethal effects),^9^, ^10^ ultimately jeopardizing biological control. A major symptom of insecticide‐mediated reductions in biological control is the sudden occurrence of otherwise innocuous or sporadic pests that are released from natural control, commonly referred to as secondary pest outbreaks.^7^, ^11^, ^12^ For example, insecticide use can increase populations of the two‐spotted spider mite (*Tetranychus urticae* Koch),^13^, ^14^ a globally important pest in many cropping systems.^15^ Similar effects of insecticide use are also documented for other key pests such as aphids.^16^, ^17^, ^18^ The mechanism underlying positive effects on pest populations is often assumed to be interference with natural enemies, but insecticides can also benefit secondary pests through other mechanisms (e.g., hormesis^14^—enhanced reproductive rate,^19^, ^20^ reduced generation time,^21^, ^22^, ^23^ female‐biased sex ratios,^22^ suppressed plant defenses^24^). Consequently, insecticide regimes targeting primary pests can inadvertently lead to secondary pest outbreaks, particularly for small arthropods with high reproductive rates (e.g., mites, aphids) that rapidly take advantage of enemy‐free space.^25^, ^26^

Aside from insecticide use, other aspects of crop management also impact pest prevalence and damage. Cover crops, for instance, are used in a wide variety of production systems with both indirect and direct effects on pest densities. Cover crops improve habitat for natural enemies by providing resources such as structural refuge and alternative food, including prey, hosts, nectar and pollen.^27^, ^28^, ^29^ As a result, cover crop use can enhance biological control, resulting in indirect negative effects on pest populations. However, cover crops can also serve as a ‘green bridge’ worsening pest pressure, resulting in direct positive effects on pest populations. A green bridge may allow pest populations to build‐up early in the season and migrate into the cash crop.^30^, ^31^ For example, both spider mites and aphid species can use vetch (e.g., *Vicia sativa* L.) as a host plant from which they move into the cash crop.^30^ In contrast, other cover crops suppress spider mites (e.g., *Festuca arundinacea* Schreb.^32^) and aphids (e.g., cereal rye, *Secale cereale* L.^33^) or have neutral effects (e.g., cereal rye and sunn hemp (*Crotalaria juncea* L.) on spider mites^34^, ^35^ and cereal rye on aphids^36^). In consequence, cover crop selection is critical to understanding pest population dynamics, but their impacts are unpredictable due to the potentially opposing effects of cover crop use on pest pressure (i.e., negative indirect effects *via* biological control *vs*. positive direct effects *via* green bridge).^30^, ^37^ Other mechanisms potentially contributing to variable pest pressure in cover cropped systems include the resource concentration hypothesis, which predicts lower pest damage in more diverse plantings due to disruptions in the ability of pests to locate and colonize the crop.^38^

In this study, we used seedless watermelon (*Citrullus lanatus* (Thunb.) Matsum. & Nakai) to understand how conventional crop management practices adopted by growers impact biological control and secondary outbreaks of spider mites and melon aphids (*Aphis gossypii* Glover). In standard insecticide programs in watermelon, broad‐spectrum pyrethroid and neonicotinoid insecticides are repeatedly applied to control the primary pest—striped cucumber beetle (*Acalymma vittatum* (Fabricius)).^39^ Spider mites and melon aphids have traditionally been considered secondary pests, but concern about spider mites is growing among watermelon producers due to resistance to miticides.^40^ Additionally, grass cover crops (predominantly cereal rye; henceforth, rye) are extensively used in watermelon as a nurse crop to protect seedlings against sand‐blasting damage.^41^ To generate this effect, rye rows are interplanted with crop rows; thus, rye is not terminated at planting like in many systems and remains present throughout the watermelon growth period. Despite the widespread cultivation of rye (i.e., >75% of large‐scale watermelon fields in our area interplant rye as a cover crop; I. Kaplan, *unpublished data*), growers have implicated rye in outbreaks of spider mites.^42^ Although the effects of rye cover cropping on multiple pests have been investigated in major row crops like corn (*Zea mays* L.), soybean (*Glycine max* (L.) Merrill) and cotton (*Gossypium hirsutum* L.),^33^, ^43^, ^44^ there is no scientific evidence indicating that rye presence contributes to spider mite outbreaks in watermelon or other fruit and vegetable crops.^34^

We combined 2 years of data from on‐farm field observations and experimental plot studies to test the individual and combined effects of insecticide use and rye presence on the primary pests, secondary pests and natural enemies. We hypothesized that conventional insecticide programs will reduce densities of the primary pest (striped cucumber beetles) and natural enemies, while increasing densities of secondary pests (spider mites, melon aphids). We further hypothesized that rye would cause a ‘spillover over’ effect, increasing the abundance of spider mites on neighboring watermelon vines. As a result, spider mite densities are expected to be the highest in watermelon fields both treated with insecticide and bordered by rye (i.e., the standard approach used on most commercial farms). Finally, due to trade‐offs in pest pressure, we hypothesized that watermelon yields will not differ across insecticide regimes and rye presence.

## 2 MATERIALS AND METHODS

### 2.1 On‐farm survey

To evaluate arthropod responses to rye use on working farms, surveys were conducted in Southwestern Indiana, USA in 2021 and 2022 on 10 and 15 commercial fields, respectively. In each year, we collaborated with five growers and scouted two (2021) or three (2022) of their fields. Each field represented one of the three levels of rye cover crop intensity: (i) no rye (0% rye presence in the field; 0:1 ratio of rye to watermelon), (ii) one rye row to every three watermelon rows (25% rye presence in the field; 1:3 ratio) and (iii) one rye row every other watermelon row (50% rye presence in the field; 1:1 ratio) (Fig. S1(A)–(C)). In total, eight fields had 0% rye, 10 had 25% rye and seven had 50% rye (Table S1). Rye intensity was not experimentally assigned (i.e., we did not randomly impose rye treatments to fields) and, thus, rye use was determined by growers in each field. Watermelon fields were separated by at least 2 km within each year, measured, on average, 16.3 ha ± SD 8.1 (range 6.9–42.9 ha) and were managed conventionally (i.e., not organic); further details in Table S1.

We recorded the density and diversity of pests and natural enemies (the latter only in 2022) on watermelon plants weekly from May (transplant) to mid‐August (harvest). Arthropods were recorded by visually scouting three (in 2021) and two (in 2022) randomly selected watermelon plants per acre (i.e., 7.5 and 5 plants per ha). Scouting consisted of (i) visually inspecting all plant parts (leaves, stems, flowers) for large arthropods (e.g., cucumber beetles, lacewings) for ~30–60 s; we also recorded arthropods that were on the beds under the plants (e.g., ground beetles) and, (ii) examining three leaves from each crown on each scouted plant to record small arthropods (i.e., spider mites and melon aphids) for ~30–120 s. Individual watermelon plants were scouted early in the season; however, as plants grew and vines entangled, we defined our scouting area as ~1 m^2^. Scouting was done between 07:00 and 13:00, alternating between fields within a farm.

We recorded (i) cucumber beetles, in which we combined striped and spotted cucumber beetles (*Diabrotica undecimpunctata* Mannerheim), (ii) spider mites and (iii) melon aphids. We also recorded squash bugs (*Anasa tristis* (De Geer))^45^ and thrips (Thysanoptera)^46^ but due to low numbers, these pests were excluded from analysis. Natural enemies recorded in 2022 were categorized into seven groups: (i) lady beetles (Coleoptera: Coccinellidae), (ii) ground and rove beetles (Coleoptera: Carabidae and Staphylinidae), (iii) lacewings (Neuroptera: Hemerobiidae and Chrysopidae), (iv) hover fly larvae (Diptera: Syrphidae), (v) true bugs (Hemiptera: Anthocoridae and Reduviidae), (vi) spiders (Araneae) and (vii) parasitoid wasps (Hymenoptera). No predatory mites (e.g., *Amblyseius* spp.) or spider mite specialized lady beetles (e.g., *Stethorus* spp.) were observed. We also recorded hover fly adults as a proxy for their larval predatory stage due to the low number of larvae observed, but we did not consider this group as a natural enemy since adults are not predators. We recorded individuals in all predatory stages (e.g., lady beetle larvae and adults; Table S2) and only predatory taxa (e.g., we recorded the hover fly *Toxomerus* spp. but not *Eristalis* spp.).

### 2.2 Research‐plot trial

#### 2.2.1 Study location, treatments and experimental field design

This trial was conducted in Indiana, USA at three Purdue University research farms (Throckmorton Purdue Agricultural Center, TPAC; Southwest Purdue Agricultural Center, SWPAC; and Southeast Purdue Agricultural Center, SEPAC). In 2023, we used the TPAC location and in 2024, we used all three research farms (henceforth, sites). This resulted in 4 year‐site combinations.

We tested the effects of insecticide management combined with the use of rye as a cover crop on pest management and watermelon yields. Each of the two factors included two levels, resulting in four treatments: (i) standard insecticide + rye presence, (ii) standard insecticide + rye absence, (iii) threshold insecticide + rye presence, and (iv) threshold insecticide + rye absence. The standard insecticide treatments (i and ii) consisted of calendar‐scheduled insecticide applications (see Section 2.2.3.), while in the threshold insecticide treatments (iii and iv), we followed an integrated pest management (IPM) approach, only applying insecticides when cucumber beetles exceeded the economic threshold of five individuals per plant.^3^, ^25^, ^47^ The rye presence (i and iii) or absence (ii and iv) treatments consisted of the use or not of a rye row as a cover crop.

For each year‐site combination, we used a single field, which included 20 plots and used a randomized complete block design to allocate the four treatments (Fig. S2). All treatments were replicated five times per field (i.e., five plot replicates per treatment each year and site; 20 replicates per treatment across all 4 year‐sites). All plots were mulched with black plastic, irrigated with drip tape and planted with 12 seedless triploid watermelon plants, cultivar ‘Fascination’, except for 2024 SEPAC, where we used ‘Powerhouse’ due to poor germination of Fascination seeds (further details in Fig. S2). Seedless watermelon seedlings were interplanted with ‘SP‐6’ diploid pollinizer plants at a 3:1 ratio, planted with a 0.6 m within‐row spacing and 1.2 m between‐row spacing between late May and early June each year. All plots were managed using standard production practices for growers in our region, including the use of fungicides and herbicides to control plant diseases and weeds, respectively.^48^ In 2023, plots measured 3.7 m length × 4 m width but in 2024, we increased the size to 5.5 m length × 4 m width, allowing a 0.9 m length buffer at either end of the plots. Plants, fruits, and arthropods in the buffer zones were not assessed.

#### 2.2.2 Rye establishment

In spring (April 21) 2023, we manually broadcasted rye longitudinally into ~0.6 m‐width rows in the rye presence treatments after light soil cultivation at a rate of 158.75 kg ha^−1^. However, rye requires vernalization over winter to reach full growth and biomass^49^ and consequently, it did not fully mature in 2023. For 2024, we drill‐seeded the rye at a rate of 102.05 kg ha^−1^, covering all the area in the plots on September 26 (SWPAC), October 2 (TPAC) and October 27 (SEPAC) 2023. All rye, except the rye rows, was mown and the soil cultivated prior to bed making. Rye rows measured ~0.6 m wide and were located between the two beds within each plot (Fig. S2) to mimic current grower practices in Indiana. We applied herbicides (a mix of flumioxazin—Chateau® and S‐Metolachlor—Dual Magnum® at label rates) to the bare ground soil on either side of the beds to suppress weeds in late May in 2024 at all three sites; no herbicides were used in 2023. All weed suppression afterwards was done manually. Uncultivated areas around the plots were frequently tilled when weeds started to grow.

#### 2.2.3 Insecticide applications

The standard insecticide treatment consisted of an initial application of the neonicotinoid imidacloprid (Dominion® 4 L) to the seedlings at planting followed by pyrethroid (Permethrin, Loveland Products, Inc.) foliar applications every 2‐weeks, which started 5–6 weeks after the imidacloprid application. Prior studies demonstrate that this is a typical insecticide regime used by many commercial watermelon producers.^26^, ^39^ At TPAC, we used a 300‐gallon (1,135.6 l) multiple point boom, vegetable sprayer selecting what nozzles (TeeJet® XR 8004‐VS) were open according to insecticide treatment, whilst at SWPAC and SEPAC, we used backpack sprayers with TeeJet® 8002VS on 38.1 cm spacing and TeeJet® AIXR 110015 on 50.8 cm spacing nozzles, respectively.

In 2023, seedlings in the standard insecticide treatments were manually drenched within 24 h after planting with 3.79 L per plant (recommended imidacloprid application rate: 0.20 mL L^−1^ of water), whereas seedlings in the threshold insecticide treatment received 3.79 L of water. We drenched the plants with 3.79 L per plant to avoid seedlings from being desiccated at the early development stage, but this resulted in an over application. To prevent an over application in 2024, we drenched the seedlings by applying imidacloprid when still in the 50‐cell seeding trays. We watered the seedlings ~12 h prior to planting with 1.89 L (same application rate as in 2023); seedlings in the threshold insecticide treatments received an equivalent amount of water. Pyrethroid applications were similar across years and sites using the recommended application rate at 584.61 mL ha^−1^.

In the threshold recommendation treatment, insecticide applications were based on whether cucumber beetles exceeded economic thresholds (five individuals per plant) determined by weekly scouting, in which case, we used the neonicotinoid acetamiprid (Assail® 70 WP at a rate 295.73 mL ha^−1^). This only happened once in 2023 TPAC 4 weeks after planting due to a combined striped cucumber beetle and melon aphid outbreak. This application was also done in the standard insecticide treatments. In 2024, the threshold treatments received no insecticide applications.

#### 2.2.4 Spider mite infestations

To ensure spider mite presence in the study, we manually infested all plots using spider mites initially collected from cucurbit fields in 2021 combined with individuals from a colony maintained at Cornell University for 5+ years and subsequently reared in the laboratory on bean (*Phaseolus lunatus* L.) plants. The laboratory colony was refreshed with wild individuals collected from watermelon plants at SWPAC for use in 2024. Spider mites were kept in insect rearing cages (BugDorm‐4F4545; dimension, 47.5 cm^3^; mesh size, 160 μm aperture) at Purdue University at 24.7 °C (± SD 4.3), 45.6% rh (± SD 7.8) (data logger, EasyLog EL‐USB‐2) and with a photoperiod of 16:8 h (L:D). We infested the watermelon plants by evenly placing infested bean leaves with spider mites in direct contact with watermelon leaves. We released spider mites in three rounds at TPAC in June, July and August 2023 and 2024 and two at SWPAC and SEPAC in June and July 2024. Additionally, at each of the three sites in May 2024, we released spider mites on the rye rows (i.e., only in the rye presence treatments) prior to planting the seedlings, allowing an opportunity for the green‐bridge mechanism to operate by building early‐season populations on the rye cover crop. On each round, we released ~2,000 and ~1,300 spider mites on the 12 watermelon plants per plot in 2023 and 2024, respectively, and ~1,500 spider mites per rye row in 2024. The number of spider mites released was estimated by sacrificing 10 in 2023 and six in 2024 bean plants for each round using the ethanol washing method and then counting the number of individuals.^50^

#### 2.2.5 Pest and natural enemy scouting

We recorded the density and diversity of arthropods as in the on‐farm survey, i.e., weekly from May (1 week after transplant) to August (the second to last harvest week). Arthropods were recorded by visually scouting two (in 2023) and three (in 2024) randomly selected watermelon plants (including three leaves per plant) per plot. Scouting was also the same, between 07:00 and 13:00, alternating between plots (mean environmental conditions ± SD–24.7 °C ± 3.3 and 68.4% rh ± 11.7; Kestrel 5000). The rye rows were also inspected for spider mites after infestation in 2024, but none were found. Pests recorded included (i) cucumber beetles (striped + spotted), (ii) spider mites and (iii) melon aphids, while natural enemies were also recorded and categorized into the same groups as in the commercial fields.

#### 2.2.6 Fruit evaluation

Watermelons were harvested between mid‐August and early‐to‐mid‐September for five consecutive weeks in 2023 and 4 weeks in 2024. The number of watermelons per plot was recorded and the individual watermelon weights measured. Additionally, insect damage on the watermelons was recorded since it can affect marketability.^51^ We recorded rind damage (caused by cucumber beetles) as the percentage cover of damage on the surface of the watermelons^51^ and honeydew (caused by melon aphids) as presence/absence^52^ in 2024 at TPAC and SEPAC during all harvests, except for honeydew which was not recorded at TPAC during the first harvest.

#### 2.2.7 Economic analysis

We used yield data to conduct a baseline economic assessment of insecticide programs across treatments (see Table S3). We used commercial values of the active ingredients and calculated the cost of individual applications per ha. We then calculated the crop revenue by multiplying the total kg harvested by the watermelon retail price of $0.16 lb^−1^ ($0.07 kg^−1^). Additionally, we considered two potential applications that growers would have made against melon aphids and spider mites. Finally, we removed the economic loss to rind damage and honeydew contamination.

### 2.3 Statistical analysis

All analyses were performed with the software R (version 4.4.2)^53^ using Generalized Linear Mixed‐Effects Models (GLMMs) and Linear Mixed‐Effects Models (LMMs) (‘lme4’ package^54^). Significant differences in the on‐farm survey were explored with Tukey *post‐hoc* tests (‘multcomp’ package^55^) with Holm‐Bonferroni corrections. Visualization was done using the function ggplot (‘ggplot2’ package^56^). To analyze pests and natural enemies, we used GLMMs with negative binomial distribution (function = glmer.nb) (model example in Table S4).

For the on‐farm survey, we specified rye level and year as fixed factors and field and week nested within year (the latter when applicable) as random factors. Year was included as a fixed factor to investigate potential environmental variation. We compared models with and without interaction and selected the most parsimonious model using the Akaike's Information Criterion (AICc)^57^ (‘MuMIn’ package^58^); i.e., lowest AICc (see Table S4). Random factors were added to account for potential geographical, temporal and environmental variability. We analyzed each of the three pests individually and natural enemies together (the seven taxonomic groups combined). To account for the repeated measures, pests and natural enemies were averaged among all plants or leaves scouted per field to obtain a single value per field, week and year. We first analyzed all data together (both years) as a general, overall model, and then followed this up with separate models for each year, when significant responses were detected (see Table S4).

For the research‐plot trial, we specified insecticide management, rye cover crop and year‐site as fixed factors and investigated the interactions using the AICc as above (see Table S4). Year‐site was included as a fixed factor due to (i) incomplete rye maturation in 2023 TPAC, (ii) differential timing of spider mite releases and insecticide applications and (iii) different cultivar used in 2024 SEPAC. Scouting week and plot nested within year‐site (the latter when applicable) were specified as random factors. As in the on‐farm survey, we also analyzed each of the three pests individually and natural enemies together. In addition, we also analyzed each separate natural enemy group (except hover fly larvae due to the low number of individuals) and hover fly adults (not considered a natural enemy group). This was not done with the on‐farm survey due to few individuals being recorded within each taxonomic group. Pests and natural enemies were also averaged among the two (in 2023) and three (in 2024) plants or leaves scouted per plot to obtain a single value per plot, week and year‐site. Similarly, we also first analyzed all data together (year‐site combinations) as a general, overall model, and then separate site‐years models when significant responses were detected (see Table S4).

The five models in the research‐plot trial (one combined, 4 year‐site specific when applicable) were also performed for each of the four harvest response variables—fruit set, fruit weight, rind damage and honeydew. Fixed factors included insecticide, rye and year‐site, and we investigated the interactions using the AICc as above. Random factors included harvest week and plot nested within year‐site (the latter when applicable). For rind damage and honeydew, we used two separate models as these metrics were not measured in 2023 TPAC and 2024 SWPAC. Fruit set and weight were square‐root transformed when necessary to meet normality (Table S4) and analyzed with LMMs (function = lmer), rind damage with a GLMM with negative binomial distribution whilst honeydew (present/absent) with GLMMs using binomial distribution (function = glmer, family = binomial) for the proportion of watermelons honeydew‐contaminated. Since no honeydew was recorded in the threshold insecticide treatment, we removed the insecticide factor from the models to avoid problems with the variance–covariance matrix estimation and only analyzed the rye effect. Honeydew was not observed in the fourth harvest and, consequently, removed from analysis.

## 3 RESULTS

### 3.1 On‐farm survey

**Figure 1:**
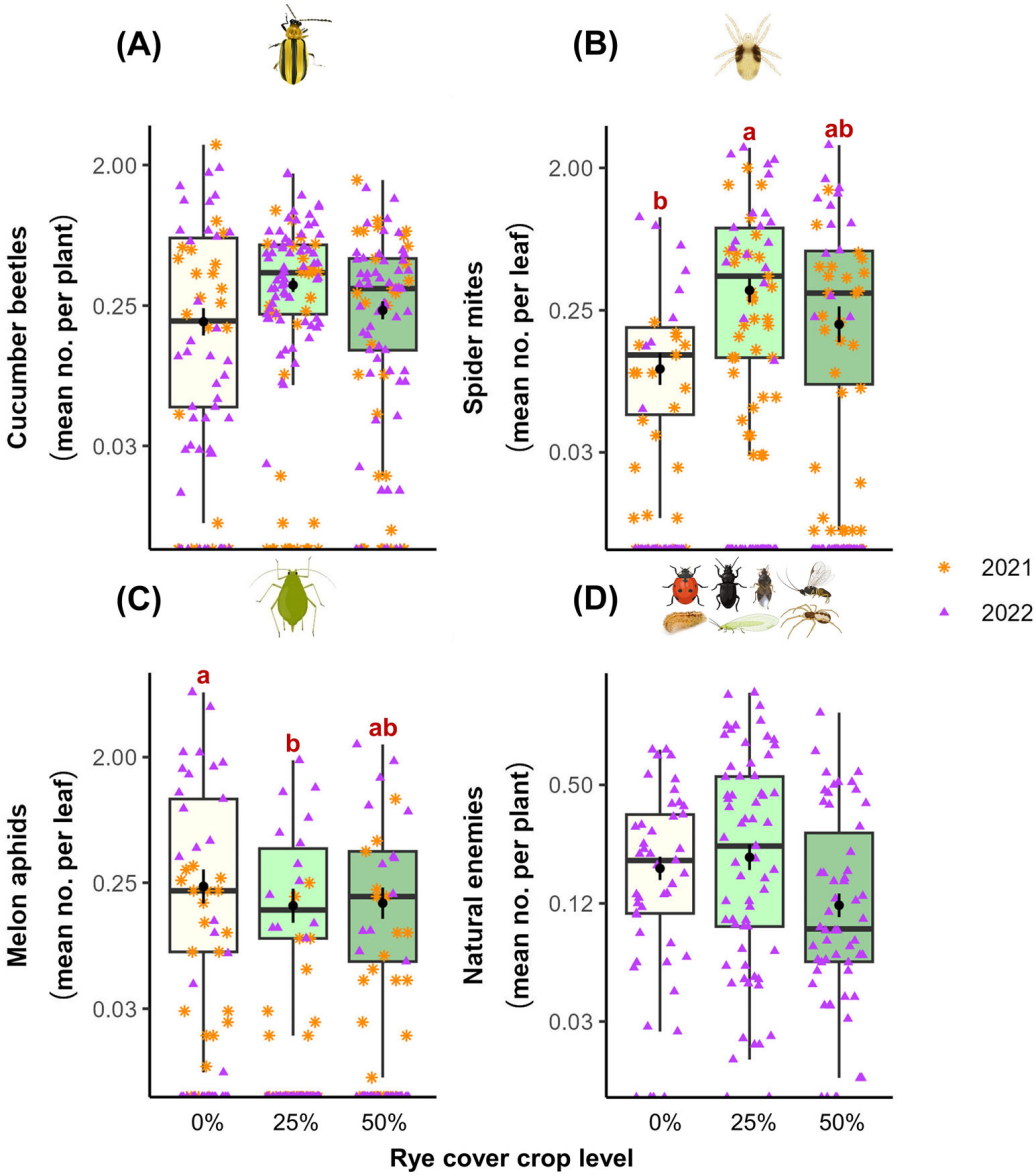
Arthropods in commercial fields. Mean ± SE of (A) cucumber beetles (striped + spotted) per watermelon plant, (B) spider mites per leaf, (C) melon aphids per leaf and (D) natural enemies per plant over the season according to rye level (0%—white, 25%—light green *vs*. 50%—dark green) and year (2021 *vs*. 2022). Y‐axes are log_2_ transformed. Superscript letters in (B,C) indicate significant differences (Tukey test, *P* < 0.05).

Overall, pest and natural enemy densities on commercial farm fields were low relative to economic thresholds (cucumber beetles, 0.4 per plant; spider mites, 0.2 per leaf; melon aphids, 0.2 per leaf; natural enemies, 0.3 per plant). Spider mites (*z* = 2.73, *P* = 0.02) and aphids (*z* = 2.51, *P* = 0.04) were significantly affected by rye level but not by year (Figs 1 and S3, Table S4). More spider mites were recorded in fields with 25% rye compared to 0% rye (Tukey test: *z* = 2.79, *P* = 0.01). In contrast, more aphids were recorded in fields with 0% rye compared to 25% rye (Tukey test: *z* = −2.38, *P* = 0.045). In both cases, this difference was only significant in the overall model, but not in each year separately. Neither cucumber beetles nor natural enemies were affected (*P* > 0.05) by rye level or year (Figs 1 and S3, Table S4).

### 3.2 Research‐plot trial

#### 3.2.1 Cucumber beetles

**Figure 2:**
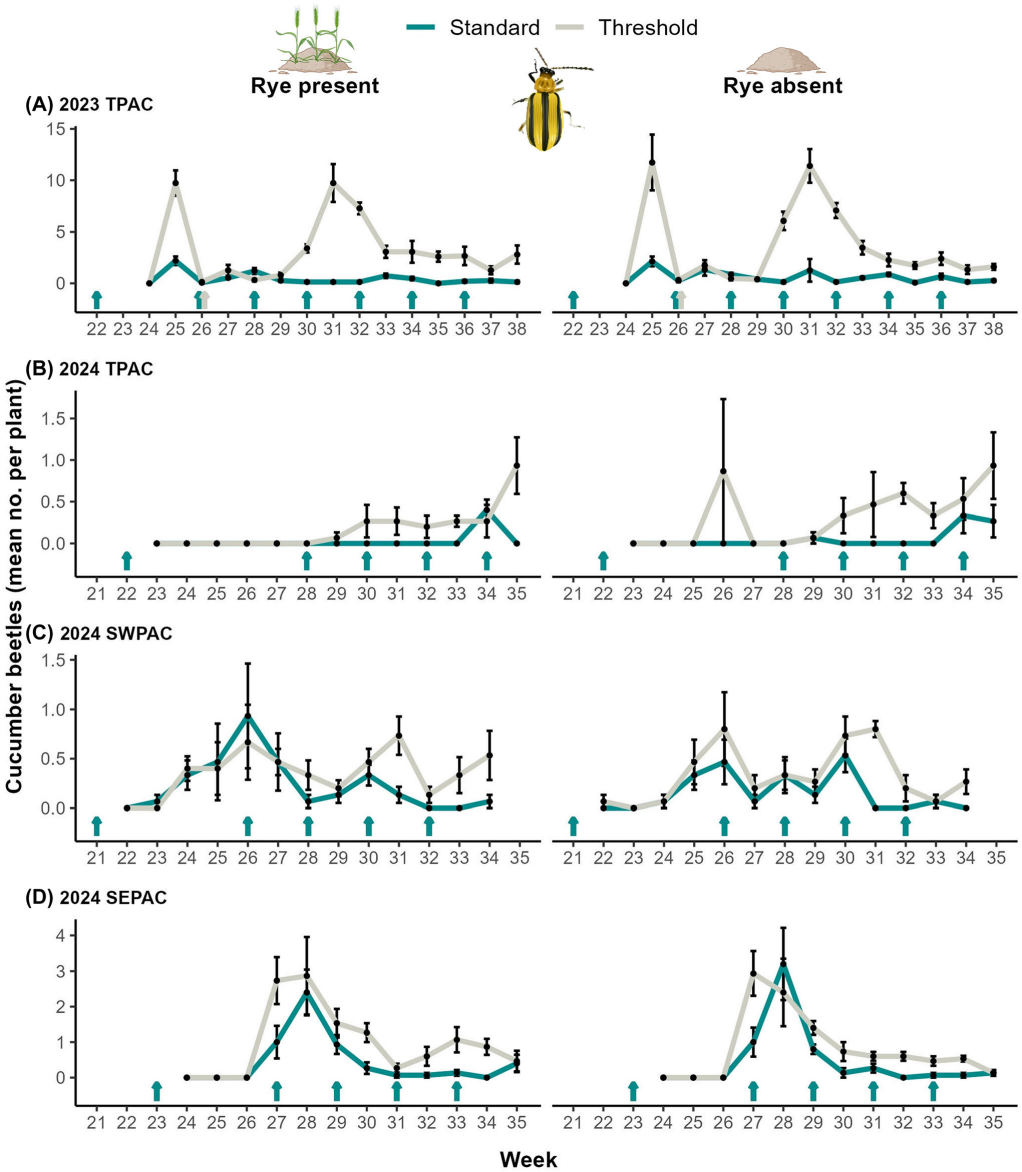
Cucumber beetles over time. Weekly mean ± SE of cucumber beetles (striped + spotted) recorded per watermelon plant according to (A–D) year‐site combination, scouting week, insecticide treatment (standard *vs*. threshold) and rye cover crop treatment (presence *vs*. absence). Cyan/gray‐colored arrows above week numbers represent insecticide applications (imidacloprid in weeks 21–23; acetamiprid in week 26 in 2023 TPAC, applied to both standard and threshold insecticide treatments; pyrethroid all other applications).

We recorded 2,315 striped and 227 spotted cucumber beetles (Table S5), which were more abundant in 2023 (mean individuals per plant: 1.9 ± SE 0.2) compared to 2024 (0.4 ± 0.03). Insecticide treatment significantly affected overall cucumber beetles with 320.9% more individuals recorded in the threshold compared to the standard insecticide treatment (*z* = 9.24, *P* < 0.001) (Figs 2 and S4). Only for cucumber beetles, the year‐site interaction was an important factor and consequently, both year‐site and the interaction between insecticide and year‐site were significant but neither rye nor interactions with rye were significant (Table S4). This pattern was consistent across all year‐site combinations except for 2024 SWPAC, where insecticide regime was not significant. The economic threshold of more than five individuals per plant was exceeded on three occasions (weeks 25, 31, and 32; Fig. 2(A)) in the threshold insecticide treatment in 2023 TPAC but not once in 2024 across any of the three sites (Fig. 2(B)–(D)).

#### 3.2.2 Spider mites

**Figure 3:**
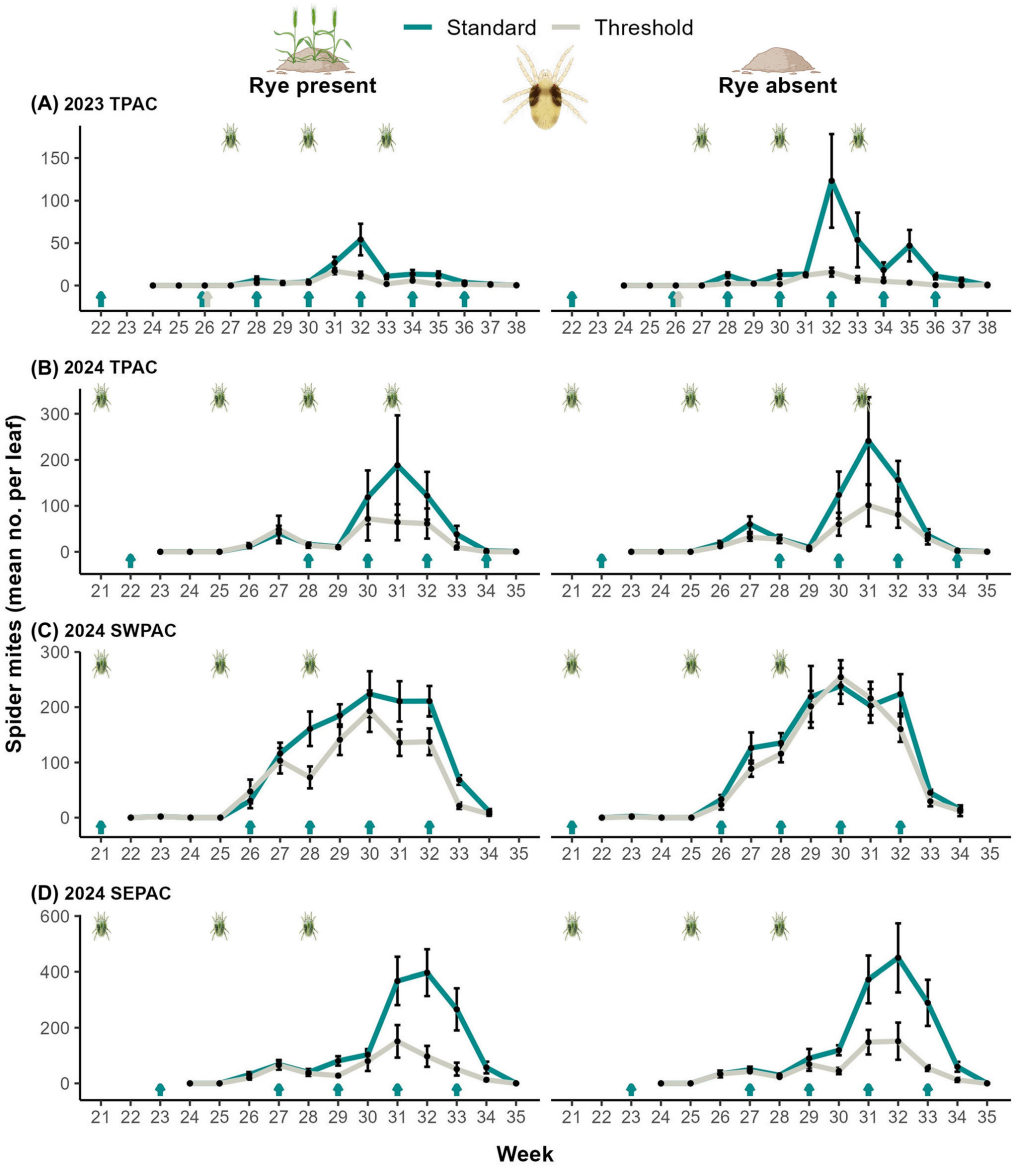
Spider mites over time. Weekly mean ± SE of spider mites recorded per watermelon leaf according to (A–D) year‐site combination, scouting week, insecticide treatment (standard *vs*. threshold) and rye cover crop treatment (presence *vs*. absence). Cyan/gray‐colored arrows above week numbers represent insecticide applications (imidacloprid in weeks 21–23; acetamiprid in week 26 in 2023 TPAC, applied to both standard and threshold insecticide treatments; pyrethroid all other applications). Spider mite icons represent releases on watermelon plants; release in week 21 in (B–D) on rye.

Spider mites were, overall, affected by insecticide applications (*z* = −3.61, *P* < 0.001) and year‐site (*z* = 13.51, *P* < 0.001), but not by rye or the interaction (Table S4). We recorded 45.5% fewer spider mites in the threshold compared to the standard insecticide treatment (Fig. S5), and this pattern was consistent throughout the study (Fig. 3). In 2024 TPAC, however, this decrease was not significant, even though treatment effects followed the same general trends as in other year‐sites (Fig. 3(B)). Additionally, in 2023 TPAC, slightly more spider mites were recorded in plots without rye (*z* = 1.96, *P* = 0.0497; mean individuals per leaf: rye present: 6.3, rye absent: 11.7).

#### 3.2.3 Melon aphids

**Figure 4:**
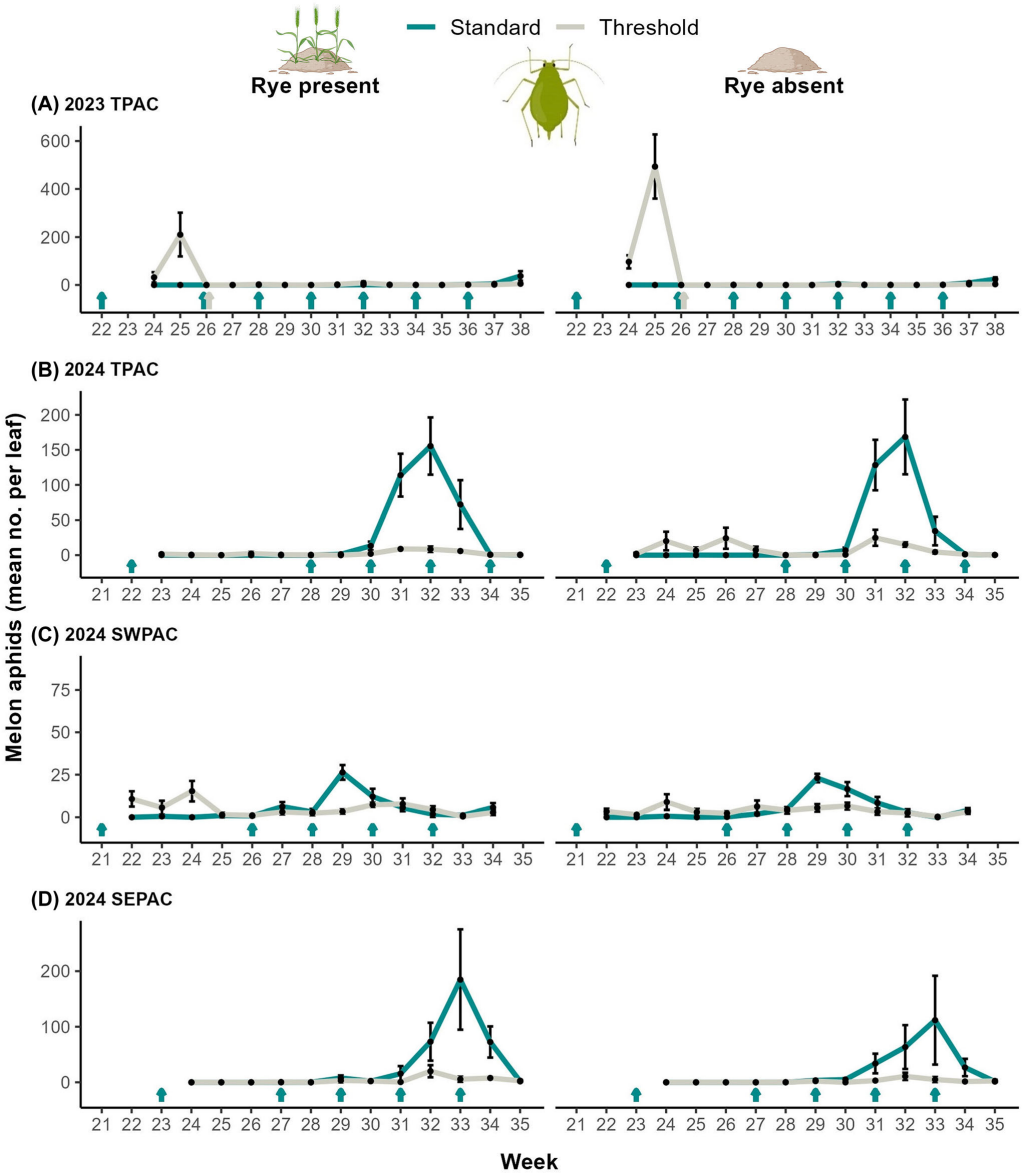
Melon aphids over time. Weekly mean ± SE of melon aphids recorded per watermelon leaf according to (A–D) year‐site combination, scouting week, insecticide treatment (standard *vs*. threshold) and rye cover crop treatment (presence *vs*. absence). Cyan/gray‐colored arrows above week numbers represent insecticide applications (imidacloprid in weeks 21–23; acetamiprid in week 26 in 2023 TPAC, applied to both standard and threshold insecticide treatments; pyrethroid all other applications).

Year‐site was an important factor for melon aphids (*z* = 3.62, *P* < 0.01; Table S4), but not insecticide treatment, rye or their interaction. However, insecticide response differed across year‐site combinations (Figs 4 and S6). In 2023 TPAC, more melon aphids were recorded in the threshold insecticide treatment, although this was driven by an outbreak occurring during the fourth week of the trial (Fig. 4(A)). In 2024, melon aphid numbers were greater in the standard insecticide treatment, significantly in TPAC and SEPAC but not significantly in SWPAC (Fig. 4(B)–(D); Table S4).

#### 3.2.4 Natural enemies

**Figure 5:**
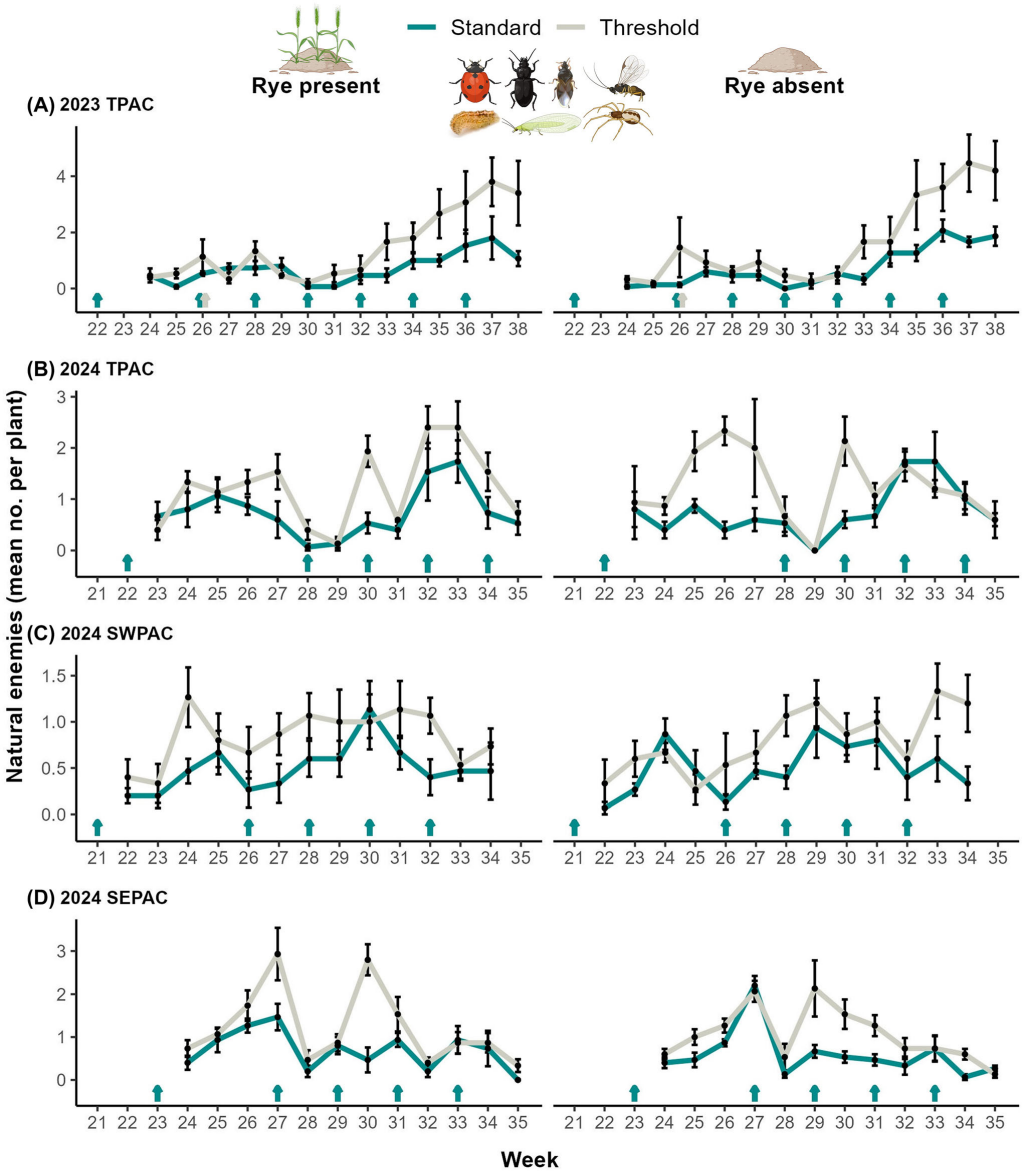
Natural enemies over time. Weekly mean ± SE of natural enemies combined recorded per watermelon plant according to (A–D) year‐site combination, scouting week, insecticide treatment (standard *vs*. threshold) and rye cover crop treatment (presence *vs*. absence). Cyan/gray‐colored arrows above week numbers represent insecticide applications (imidacloprid in weeks 21–23; acetamiprid in week 26 in 2023 TPAC, applied to both standard and threshold insecticide treatments; pyrethroid all other applications).

We recorded a total of 2,958 natural enemies with spiders as the most abundant group with 50.3% of the records, followed by lady beetles (21.0%) and lacewings (13.1%) (Table S5). Insecticide treatment had a significant effect on all natural enemies combined (*z* = 6.35, *P* < 0.001), with 82.3% more individuals in the threshold insecticide treatments. Year‐site was also an important factor (*z* = 4.03, *P* < 0.01), but neither rye nor the interaction had a significant effect (Figs 5 and S7; Table S4). This outcome was consistent across the year‐site combinations (Fig. 5). Natural enemy groups separately also followed similar numerical trends in most cases, being significantly more abundant in the threshold insecticide treatment, including lady beetles, flower + assassin bugs and spiders (along with the non‐natural enemy group hover fly adults) but they were not always significant in each year‐site (Table S4).

#### 3.2.5 Harvest–fruit set, weight, and quality

**Figure 6:**
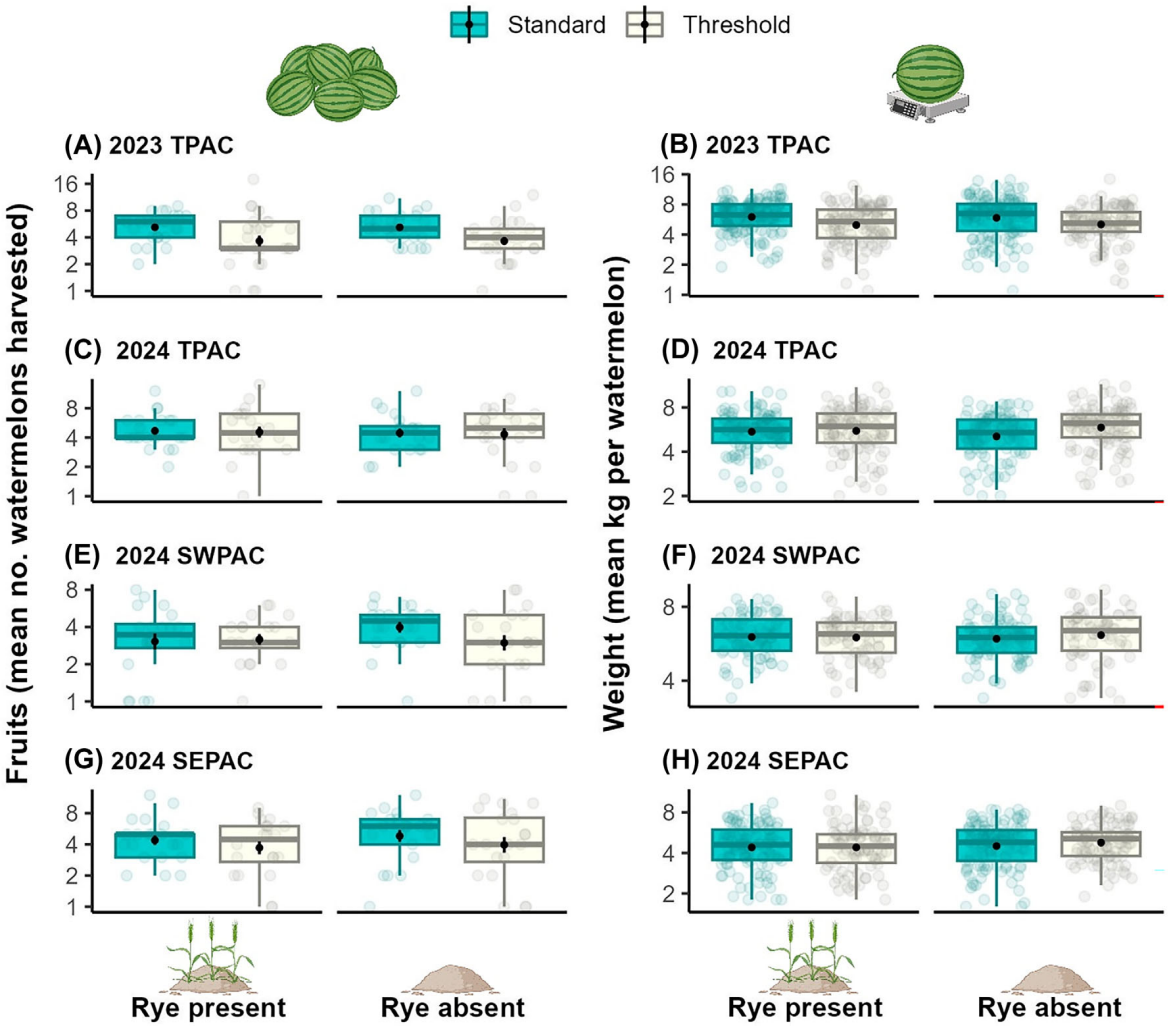
Harvest. Mean ± SE number of (A,C,E,G) watermelons harvested and (B,D,F,H) weight per watermelon per plot and week (light colored dots) according to year‐site combination, insecticide treatment (standard *vs*. threshold) and rye cover crop treatment (presence *vs*. absence). Y‐axes were log_2_ transformed.

Across all 4 year‐sites, we harvested a total of 1,618 watermelons with a total weight of 9,178.7 kg (2023 TPAC: 497 watermelons, 2,982.7 kg; 2024 TPAC: 413 watermelons, 2,389.6 kg; 2024 SWPAC: 409 watermelons, 1969.9 kg; and 2024 SWPAC: 299 watermelons, 1,836.5 kg). Across all year‐sites combined, there were no significant overall effects of insecticide, rye or year‐site on either the number of fruits harvested or their weight (Fig. 6; Table S4). This was consistent with three of the 4 year‐sites but in 2023 TPAC, we harvested 3.8% fewer watermelons (*z* = −2.10, *P* = 0.04) that weighed 15.1% less (*z* = −2.30, *P* = 0.04) with the threshold insecticide treatment.

Among the 822 watermelons assessed for rind damage (Fig. S8), only seven (one in the standard and six in the threshold insecticide treatments) had damage greater than 10% (allowed by the USDA retailer metric standards), which resulted in a loss of 0.85% (0.12% standard *vs*. 0.73% threshold) in marketability. Aphid honeydew was only present on fruits in the standard insecticide treatment plots and the 41 watermelons with honeydew contamination (out of 260 assessed) represent a 15.8% loss in marketability.

#### 3.2.6 Economic analysis

The total crop revenue was 15.2% higher in the standard insecticide program ($6,151 ha^−1^) compared to the threshold ($5,338 ha^−1^). However, using marketable yields, revenue was 5.5% lower in the standard program ($5,005 ha^−1^; threshold = $5,299 ha^−1^). Our insecticide cost was 7× higher in the standard ($298 ha^−1^) compared to the threshold ($44 ha^−1^). This cost would increase up to 13× in standard *vs*. thresholdwhen considering additional aphid‐specific and miticide applications (Table S3).

## 4 DISCUSSION

Overall, our results show that rye has little impact on pests or natural enemies, suggesting that spider mites or melon aphids are unlikely to use it as a green bridge to move into the crop. Rather, extensive use of broad‐spectrum insecticides was a critical factor in increasing both pests and decreasing natural enemies on watermelon vines.

### 4.1 Effect of insecticides on arthropods

Our data demonstrate that repeated use of neonicotinoid and pyrethroid insecticides may be unnecessary for controlling the primary pest, cucumber beetles, and applications based on IPM threshold recommendations would suffice.^25^ Throughout our study, we only sprayed a single insecticide application in the threshold insecticide plots due to a combined outbreak of striped cucumber beetles (mean of 10.7 per plant) and melon aphids (mean of 351.9 per leaf). Additionally, the difference in cucumber beetle density between years further emphasizes the importance of IPM, since in 2024, cucumber beetles were scarce and never reached the economic threshold of five individuals per plant (see Fig. 2) to justify any calendar‐scheduled insecticide application.

While broad‐spectrum insecticides were effective at controlling cucumber beetles, the increase of both spider mites and melon aphids in the standard insecticide treatment suggests that neonicotinoids and pyrethroids have limited success at controlling secondary pests and can, in turn, flare their populations.^12^, ^19^ Neonicotinoids can have beneficial effects on spider mite populations,^24^ which might have contributed to the greater populations we recorded in the standard insecticide treatment. Neonicotinoids can impair the transcription of defensive enzymes in plants, which function to protect against herbivores, resulting in an amplified susceptibility to pests.^24^, ^59^ This disruption in plant defenses might have favored spider mites in the standard insecticide plots treated with imidacloprid. Because our standard insecticide treatment combined two active ingredients to mimic grower practices, we are unable to differentiate the relative importance of neonicotinoids *versus* pyrethroids in causing spider mite outbreaks.^22^, ^24^

The greater abundance of both spider mites and melon aphids in the standard insecticide treatment was also likely caused by a disruption in natural enemy dynamics, in combination with the direct effects of insecticides. Natural enemy reduction due to pyrethroid applications is a frequently reported outcome in the literature.^60^ IPM can help, thereby, protect natural enemies and keep secondary pests from outbreaking. Indeed, a recent study also shows that threshold‐based insecticide recommendations in watermelon results in increased natural enemy populations and predation rates on surrogate prey.^3^ Similarly, Pecenka *et al*.^25^ only observed economically damaging populations of spider mites and melon aphids in conventionally managed compared to IPM watermelon plots. The small‐plot nature of our trial likely underestimates the positive impacts of insecticides on secondary pests since, in more realistic conditions, large field size would prevent natural enemies from rapidly recolonizing insecticide‐treated crops and result in correspondingly larger spider mite and/or melon aphid outbreaks. Future work should be conducted in large‐scale field settings, also minimizing plot proximity, to confirm the effects of the experimental treatments on pests and natural enemies.

### 4.2 Effect of rye cover crop on arthropods

A major concern of Indiana watermelon growers is spider mites using rye to move into the cash crop, but this anecdote is not supported by our data. In the on‐farm survey, spider mites were positively affected only by the intermediate rye presence (25 > 0%), but the magnitude of this effect was relatively low and at population levels that are not economically concerning for growers (mean of 0.2 per leaf). Unless outbreaking, these low spider mite levels in the commercial fields are typical and a greater presence of this pest could have helped better confirm their use of rye. Due to the high variability of spider mite outbreaks across years and locations, we artificially infested our research‐plot trial to ensure consistent pest pressure and effectively test treatments. Specifically, we infested the rye in the 2024 season before planting the watermelons to allow populations to build and spillover to young, vulnerable seedlings. This represented a natural simulation of how colonization may happen and provided an opportunity for the ‘green bridge’ mechanism to operate as intended. However, even when we directly infested rye in our research‐plot trial, we still failed to see strong evidence that rye acts as a ‘green bridge’ and, in fact, were unable to recover spider mites from rye, despite visually searching these plants even 1‐week after infestation. As an annual plant, rye begins senescing in late May/early June and it completely dries out by mid‐June.^61^ Consequently, rye may not provide resources to sustain spider mites by this time of the year. Spider mite outbreaks in commercial fields are typically reported starting in July in the Midwestern US, well beyond the end of active rye growth. Additionally, the fact that we only recorded spider mites after the first infestation on watermelon plants in 2024 at the three sites, despite having infested the rye prior to planting, suggests that rye may not be a preferable host for spider mites. During our study (June–August), weather was within the average conditions typical for the region (mean in 2022: 24.3 °C and 79.7% rh and in 2023: 25.0 °C and 79.7% rh; timeanddate.com), but warmer and drier conditions from extreme events may differently affect spider mite dynamics and promote outbreak conditions.

In our study, overall densities of melon aphids were lower in plots with rye, however this pattern was not significant in any of the year‐sites separately. Rye can disrupt melon aphid colonization of the crop by providing an alternative host plant directly competing with the cash crop through visual interference.^62^ The interaction between insecticide and rye suggests that IPM in combination with rye cover crop is the best option to control this pest. Importantly, a number of studies report enhanced biological control with rye, which may have reduced aphid numbers. Although positive effects of cover crops on natural enemies can vary depending on the landscape,^63^ cover crops generally support natural enemies.^29^ For example, natural enemies were enhanced early in the season with rye in cotton, which helped control pests.^64^, ^65^ However, our rye effect is more consistent with Koch *et al*.,^33^ where soybean aphids were reduced in soybean with rye present, but without a positive natural enemy response. Thus, rye may affect natural enemies differently depending on the cropping system in which it is used and/or the major pests that are available.^43^ Rye and other cereal species can also support different aphid species that do not use watermelon as a host plant (e.g., bird cherry oat aphid, *Rhopalosiphum padi* L.; English grain aphid, *Sitobion avenae* Fabricius),^66^ and consequently could support natural enemies via alternative prey. Alternative flowering cover crops may provide more resources to improve biological control in watermelon. For example, using red clover (*Trifolium pratense* L.) as a cover crop in cucumber (*Cucumis sativus* L.) enhanced natural enemies, while reducing striped cucumber beetles and melon aphids.^67^

### 4.3 Effect of insecticide management and rye cover crop on yield

In 2024, threshold and standard insecticide treatments produced statistically similar yields. However, threshold treated watermelons were fewer and smaller in 2023, which could have been due to the high cucumber beetle pressure in this year‐site. Leach and Kaplan^47^ did not find a reduction in yield with up to nine individuals per plant, but in our study we recorded almost 11 per plant on two occasions (weeks 25 and 31, see Fig. 2(A)). Delays in spraying on week 31 might have caused the reduced yield effect in this year‐site, which highlights the importance of following timely management interventions when using economic thresholds to avoid crop losses. Additionally, our plants were exposed to high levels of spider mites that could have contributed to the lower yield. Another potential factor contributing to lower fruit set is the presence of cucumber beetles, which can reduce pollinator visits to flowers.^68^ Cucumber beetles were abundant in weeks 31–32 and consequently, they could have disrupted bees from pollinating crop flowers, thereby intensifying the lower fruit set in 2023 TPAC. To prevent these detrimental effects, including delays in insecticide application, a more conservative economic threshold of two cucumber beetles per plant could be used, as in previous studies.^26^ Cucumber beetles can also cause rind damage and reduce marketability.^51^ Even though we only recorded rind damage at two sites in 2024, we demonstrate that when cucumber beetles are kept under economic thresholds, rind damage rarely exceeds market standards, thereby, causing negligible crop losses.^47^

### 4.4 Economic analysis

The outcomes from our baseline economic assessment depended largely on crop marketability as shown by the crop revenue before and after considering marketability. Marketability differences were mainly caused by honeydew contamination of fruits by late‐season aphid populations that rapidly increased following pyrethroid applications. Under commercial conditions, growers may have preemptively applied an additional insecticide to avert this outbreak. These crop prices should also be evaluated in light of insecticide cost differences, which were 7× higher in the standard compared to the threshold. Most of these cost differences derive from neonicotinoid applications since pyrethroids, which constituted most of the inputs, are incredibly cheap. If we assume that growers would have applied an additional aphid‐specific product and a miticide to control secondary pest outbreaks, this would more than double the standard costs (to $600 ha^−1^), resulting in a 13× increase in standard *versus* threshold. Nevertheless, the absolute value of insecticide regimes is only a fraction of crop value and thus difference in crop performance is the primary factor shaping the economics of IPM in this system. This analysis does not account for labor and production costs (e.g., crop seed, irrigation equipment, herbicides and fungicides) that ultimately determine system profitability.

## 5 CONCLUSION

Growers prioritize neonicotinoids and pyrethroids to control cucumber beetles but management for this pest clearly disrupts the natural control of spider mites and melon aphids by natural enemies. Consequently, watermelon growers could benefit from IPM programs to enhance sustainable production and minimize insecticide costs. This outcome dovetails with a growing body of literature showing that watermelon pollination is improved under IPM‐managed fields.^4^, ^25^, ^26^, ^47^ Thus, both ecosystem services (biological control + pollination) can be enhanced in this cropping system by implementing threshold‐based recommendations.

Importantly, we did not find evidence of rye supporting spider mite populations, despite anecdotal claims from most large‐scale cucurbit growers in our region that rye is used as a green bridge for spider mites to colonize the cash crop. However, rye also does not seem to support natural enemies and therefore has limited beneficial impacts on sustainable pest management by supporting biological control. We recommend the testing and potential adoption of alternative flowering cover crops (e.g., vetch, clover) that maintain the protective windbreak function of rye while simultaneously providing supplemental nectar and pollen for better natural enemy establishment.

## CONFLICT OF INTEREST

The authors declare no conflict of interest.

## Supporting information


**Data S1.** Figures.

**Data S2.** Tables.

## Data Availability

The data that support the findings of this study are openly available in Secondary‐pest‐outbreaks_Mateos‐Fierro at https://github.com/ZeusMF/Secondary-pest-outbreaks_Mateos-Fierro.
