# Supplementary material for: Insecticides outweigh rye cover crop in triggering secondary pest outbreaks
Source: Pest Manag Sci. 2025 Aug 2;81(12):7971–84. doi: 10.1002/ps.70109 (PMC12618915; doi:10.1002/ps.70109)
Supplement: Supplementary file 1 — Data S1. Figures. [file PS-81-7971-s001.pdf]

Insecticides outweigh rye cover crop in triggering secondary pest outbreaks

Zeus Mateos-Fierro, Ashley Leach, Ian Kaplan

Supplementary figures

Table of contents

Figure S1. Rye cover crop levels 2

Figure S2. Plot plan 3

Figure S3. Arthropods in commercial fields 4

Figure S4. Cucumber beetle density 5

Figure S5. Spider mite density 6

Figure S6. Melon aphid density 7

Figure S7. Natural enemy density. 8

Figure S8. Rind damage and honeydew 9

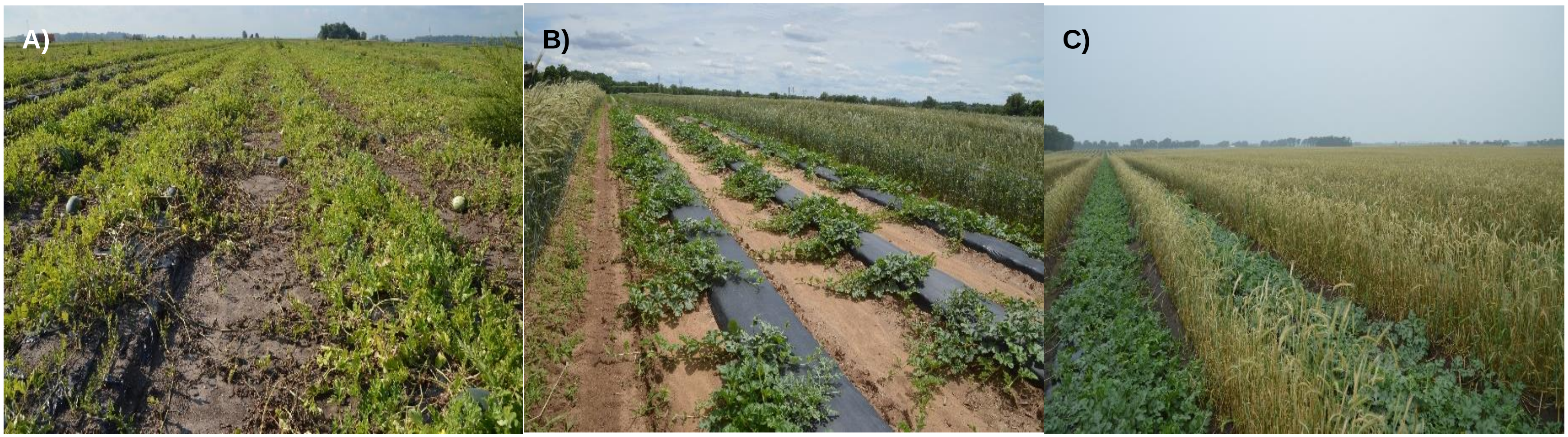

Figure S1. Rye cover crop levels. A) Watermelon field with no rye (0% rye presence in the field; 0:1 ratio of rye to watermelon), B) watermelon field with one rye row to every three watermelon rows (25% rye presence in the field; 1:3 ratio) and C) watermelon field with one rye row every other watermelon row (50% rye presence in the field; 1:1 ratio).

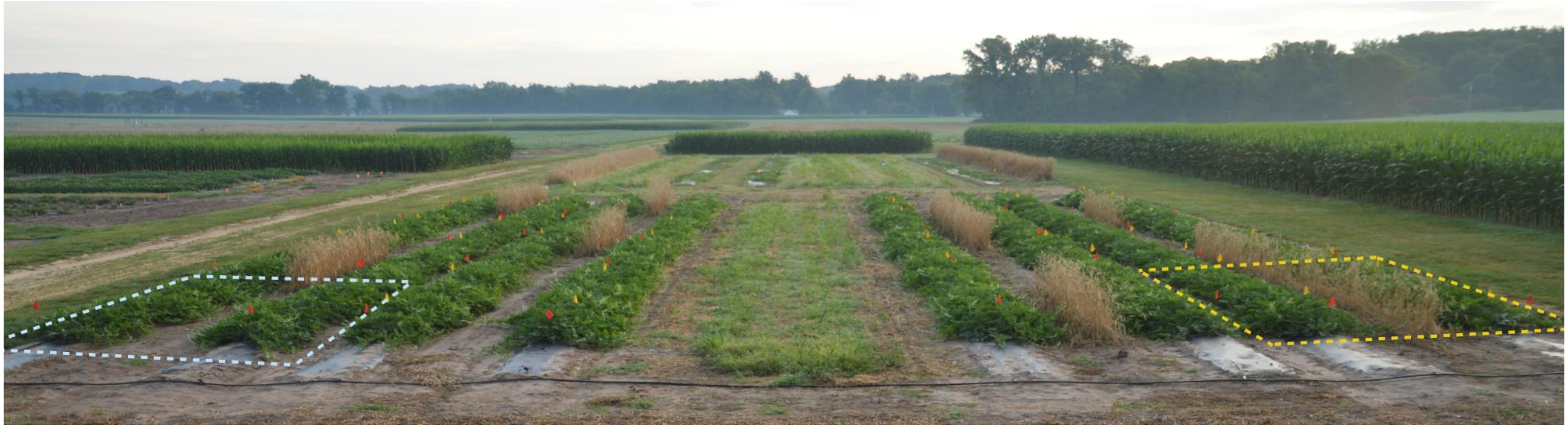

Figure S2. Plot plan. Example of one of the fields (2024 SWPAC) including the 20 plots; four treatments replicated five times. Two plots are highlighted with dashed line polygons—bottom left corner in blue rye absent; bottom right corner in yellow rye present. Red flags delimit plots including the buffer area while yellow flags within the plots exclude the buffer area. All plots were mulched with black plastic (Ginegar Plastics Inc. 1.2 m wide, 2 mm thick) and irrigated with drip tape (T-tape Rivulis, 3.8 l/min per 30.5 m, 30.5 cm emitter spacing, 0.3 mm thick). Plots included 12 'Fascination' watermelons (except for 2024 SEPAC which included 'Powerhouse') and interplanted with the pollinizer 'SP-6' at a 3:1 ratio. All watermelons were Syngenta®, USA cultivars, which we germinated from insecticide-untreated seeds. Photo taken on 08 July 2024.

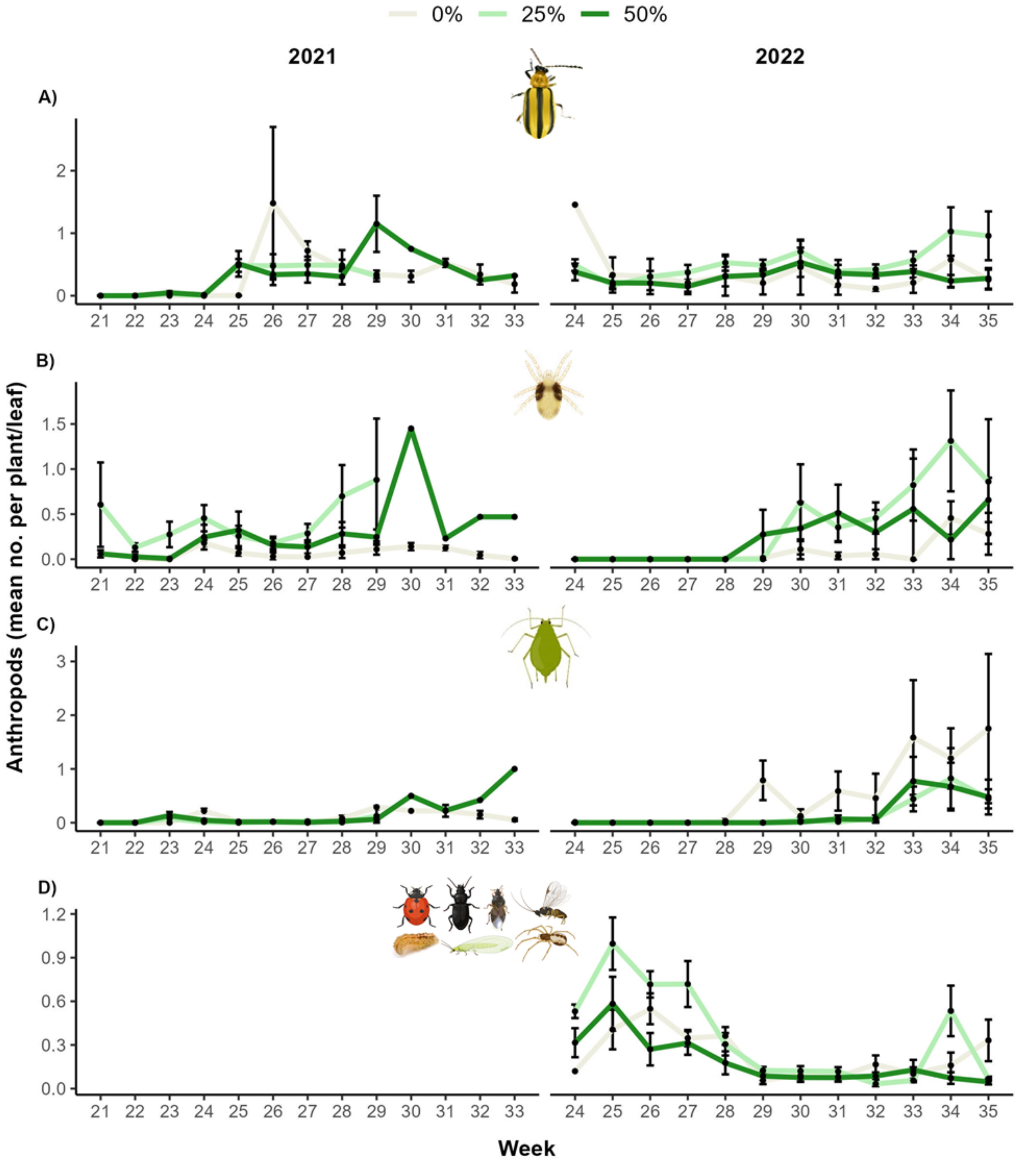

Figure S3. Arthropods in commercial fields. Weekly mean ± SE of A) cucumber beetles (striped + spotted) per watermelon plant, B) spider mites per leaf, C) melon aphids per leaf and D) natural enemies per plant recorded in commercial fields according to scouting week, rye cover crop level (0%, 25% vs. 50%) and year (2021 vs. 2022). Y-axes were $\log_2$ transformed; note differences in Y-axes. Natural enemies were not recorded in 2021.

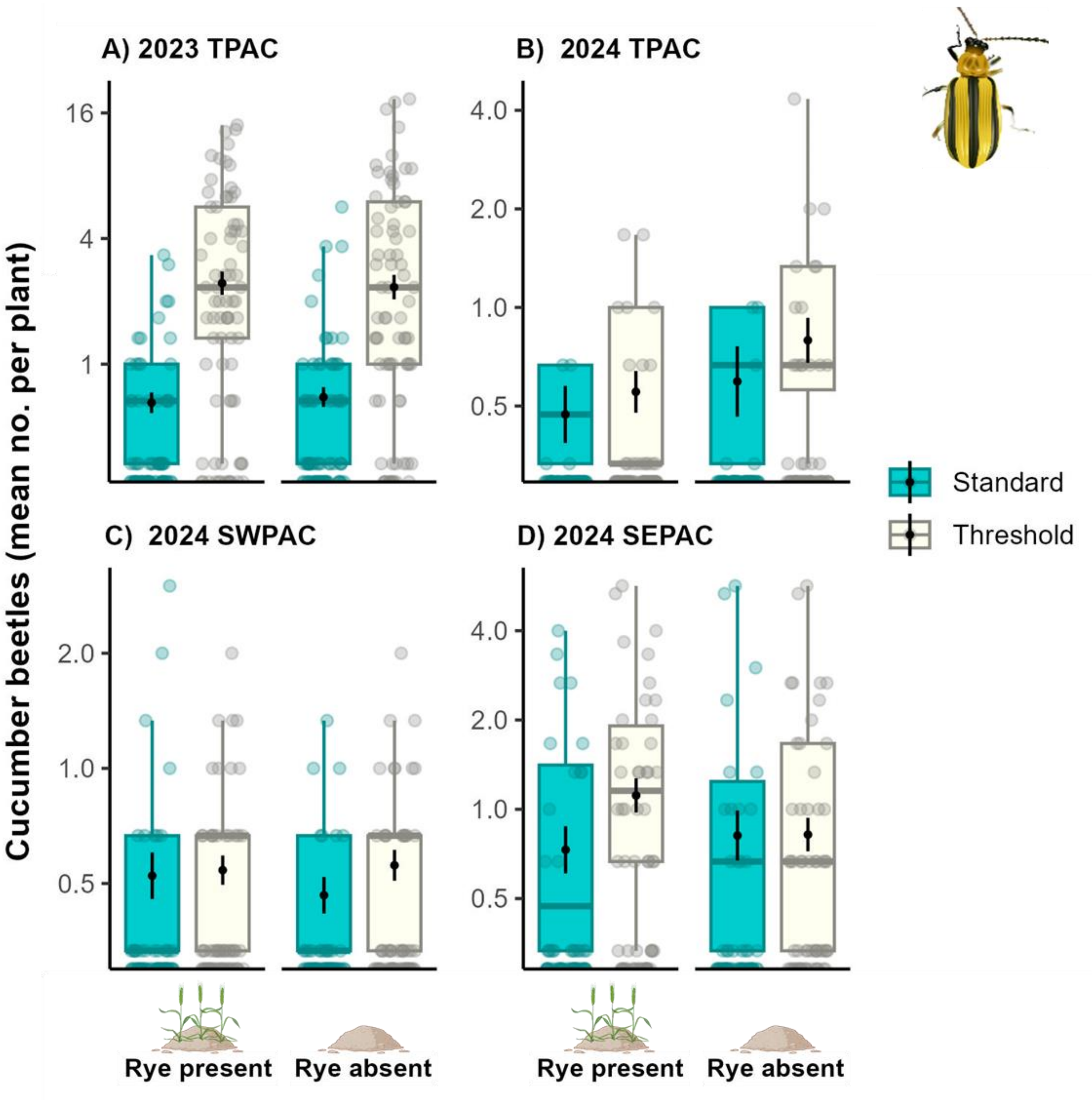

Figure S4. Cucumber beetle density. Mean ± SE of cucumber beetles (striped + spotted) recorded per watermelon plant over the season according to A-D) year-site combination, insecticide treatment (standard vs. threshold) and rye cover crop treatment (presence vs. absence). Y-axes were log_2 transformed; note differences in Y-axes. Light colored dots represent averages per plot and scouting week.

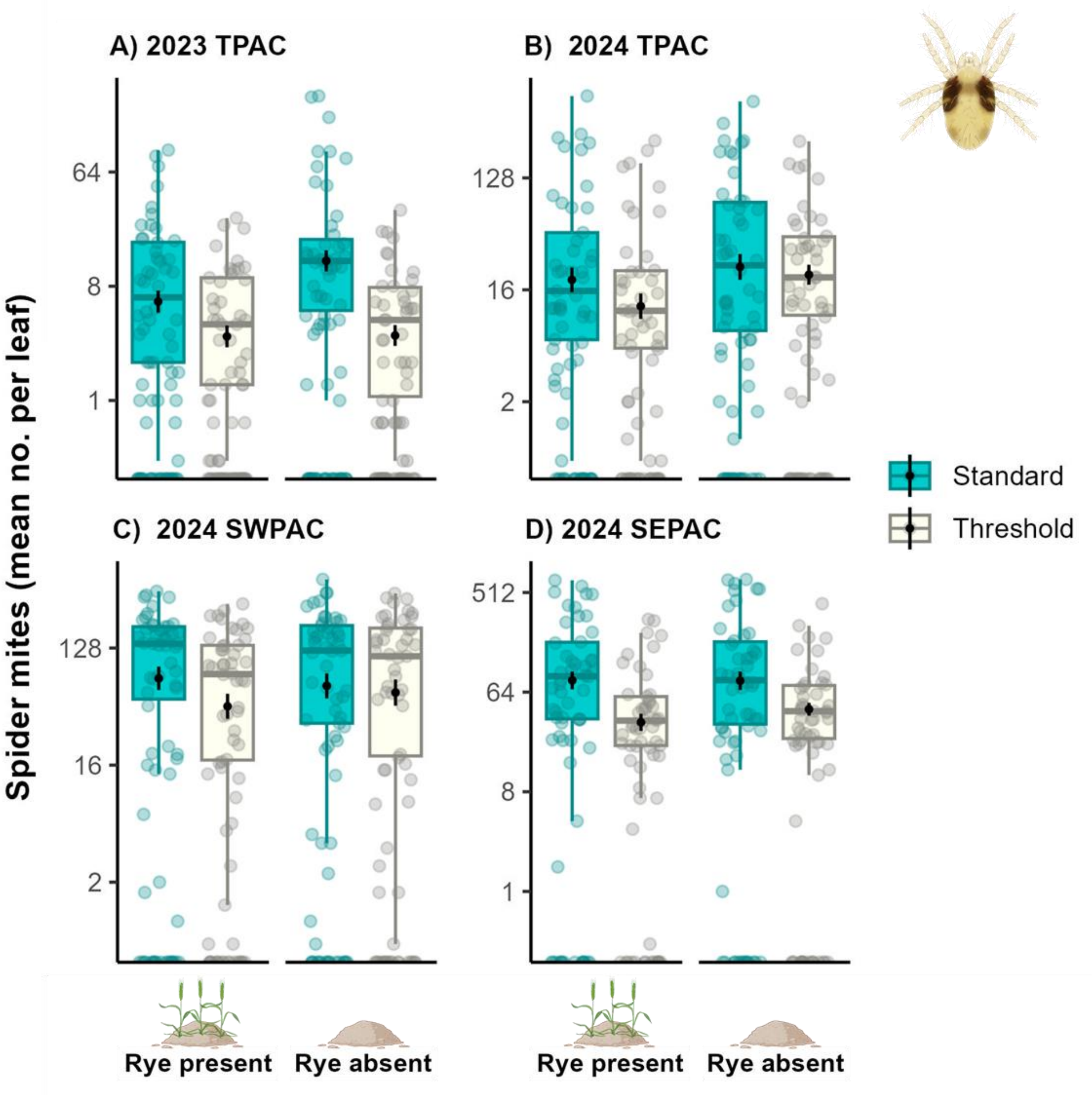

Figure S5. Spider mite density. Mean ± SE of spider mites recorded per watermelon leaf over the season according to A-D) year- site combination, insecticide treatment (standard vs. threshold) and rye cover crop treatment (presence vs. absence). Y-axes were $\log_2$ transformed; note differences in Y-axes. Light colored dots represent averages per plot and scouting week.

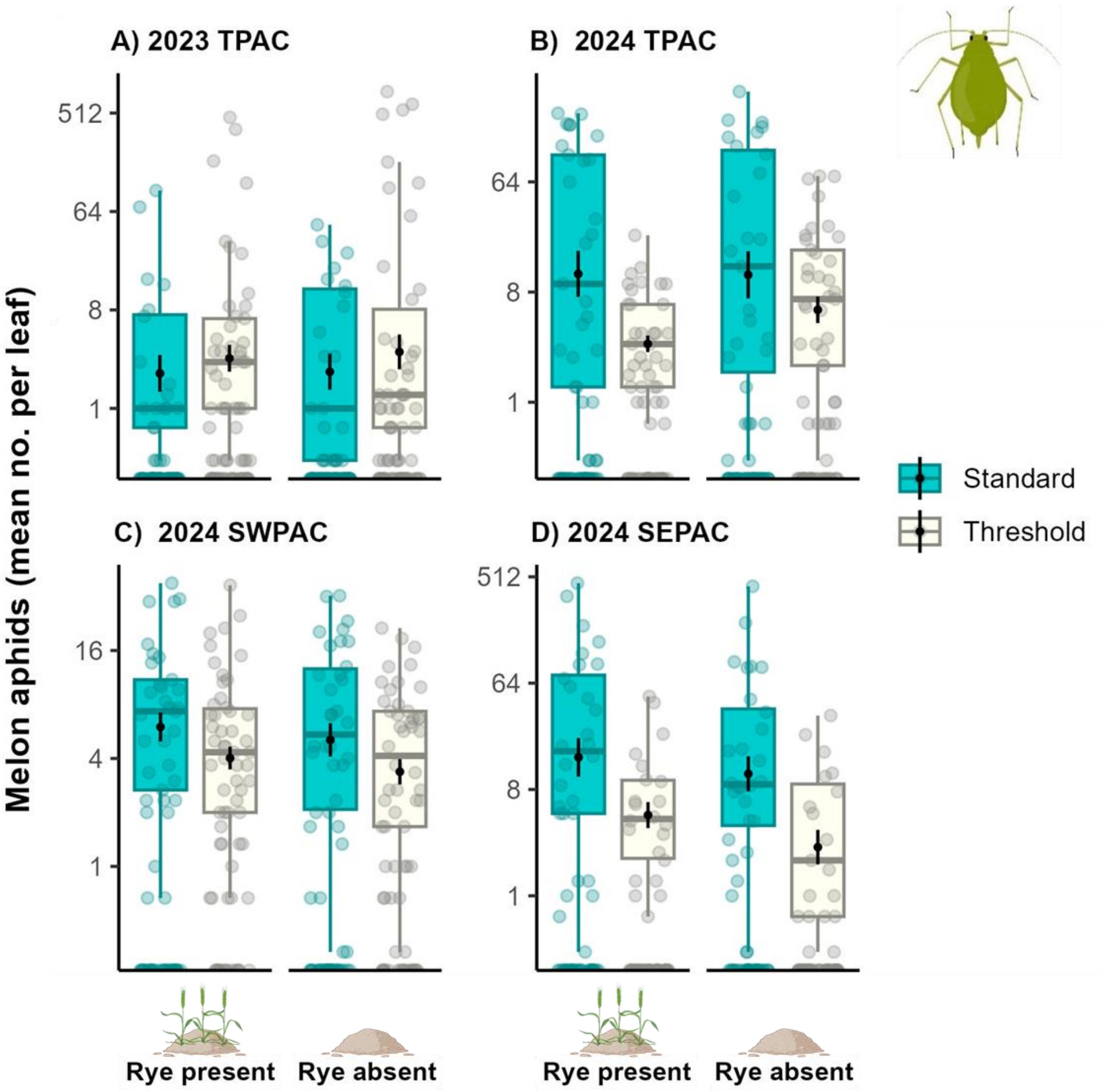

Figure S6. Melon aphid density. Mean ± SE of melon aphids recorded per watermelon leaf over the season according to A-D) year- site combination, insecticide treatment (standard vs. threshold) and rye cover crop treatment (presence vs. absence). Y-axes were log_2 transformed; note differences in Y-axes. Light colored dots represent averages per plot and scouting week.

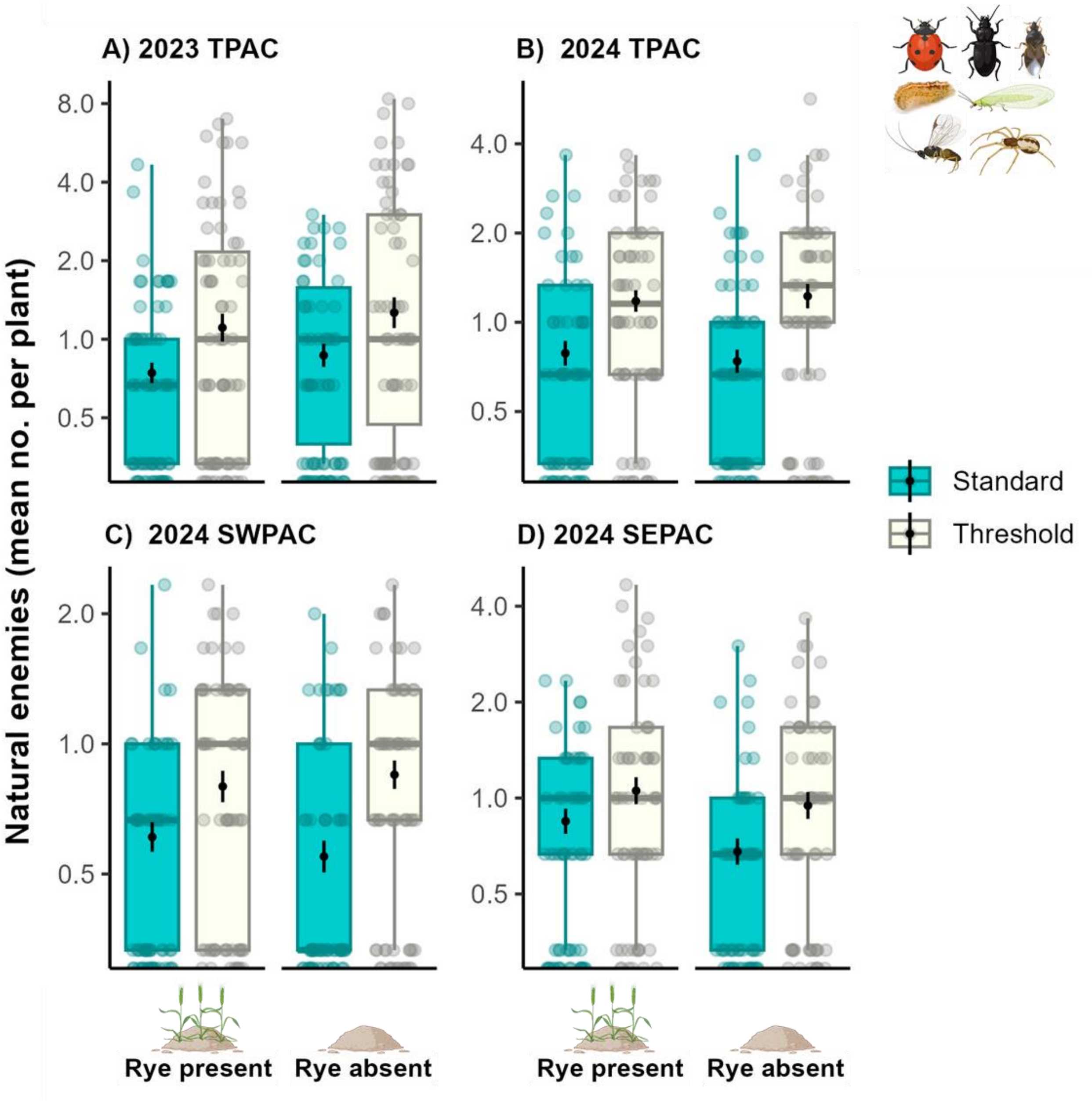

Figure S7. Natural enemy density. Mean ± SE of natural enemies combined recorded per watermelon plant over the season according to A-D) year- site combination, insecticide treatment (standard vs. threshold) and rye cover crop treatment (presence vs. absence). Y-axes were log_2 transformed; note differences in Y-axes. Light colored dots represent averages per plot and scouting week.

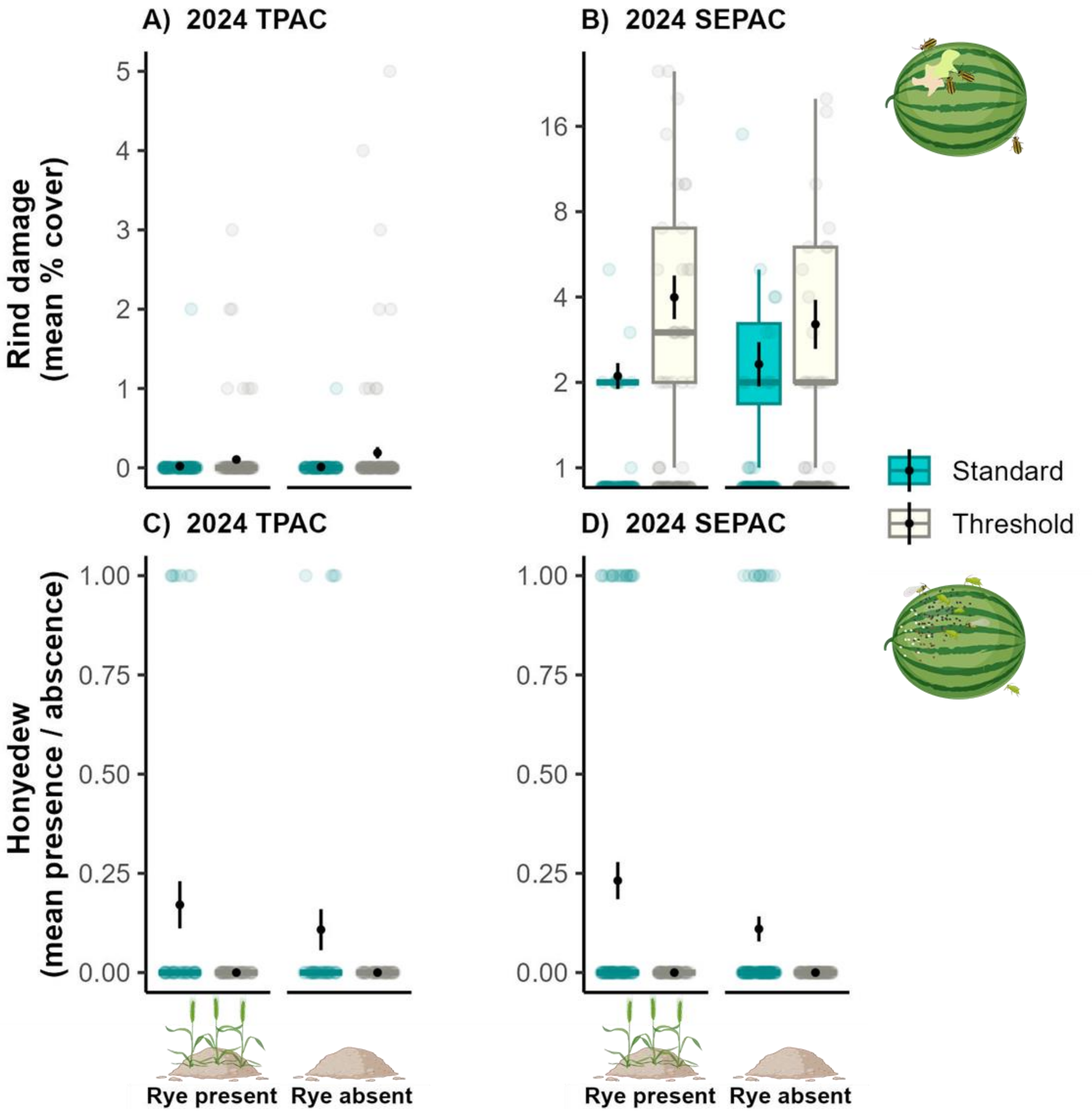

Figure S8. Rind damage and honeydew. Mean ± SE of A, B) rind damage percentage coverage and C, D) honeydew presence/absence per watermelon according to year-site combination, insecticide treatment (standard vs. threshold) and rye cover crop treatment (presence vs. absence). Honeydew was recorded in 41 out of 260 watermelons in the standard insecticide treatment vs. 0 out of 258 in the threshold insecticide treatment. Y-axes were $\log_2$ transformed in A and B; note differences in Y-axes. Light colored dots represent averages per plot and harvest week.
